# Sex Differences in the Amount and Patterns of Car-Driving Exposure in Spain, 2014 to 2017: An Application of a Quasi-Induced Exposure Approach

**DOI:** 10.3390/ijerph182413255

**Published:** 2021-12-16

**Authors:** José Mateos-Granados, Luis Miguel Martín-delosReyes, Mario Rivera-Izquierdo, Eladio Jiménez-Mejías, Virginia Martínez-Ruiz, Pablo Lardelli-Claret

**Affiliations:** 1 Department of Preventive Medicine and Public Health, School of Medicine, University of Granada, Avenida de la Investigación 11, 18071 Granada, Spain; josemateos98@gmail.com (J.M.-G.); luismiguelmr@ugr.es (L.M.M.-d.); mariorivera@ugr.es (M.R.-I.); eladiojimenez@ugr.es (E.J.-M.); lardelli@ugr.es (P.L.-C.); 2 Health Research Institute of Granada (Instituto Biosanitario de Granada), ibs.Granada, 18012 Granada, Spain; 3 Centre for Biomedical Research in Network of Epidemiology and Public Health (CIBERESP), 28029 Madrid, Spain

**Keywords:** car-driving exposure, gender differences, environmental factors, traffic epidemiology, quasi-induced exposure

## Abstract

We designed a cross-sectional study in Spain, from 2014 to 2017. Our objective was to assess sex-related differences in the amount of driving exposure of car drivers, overall and stratified by the main environment-related driving conditions. We compared the sex distribution across three populations: (1) total number of person-years aged > 18 years; (2) total number of person-years aged > 18 years holding a valid car-driving license; and (3) total number of non-responsible car drivers involved in crashes with another offending driver, stratified by different environmental variables. The quasi-induced exposure approach was applied: the non-responsible drivers were considered as representative of the entire population of drivers on the road at the place and time at which the crash occurred. We calculated the female-to-male odds ratio (OR) by comparing population 2 versus 1, and population 3 versus 2. Finally, we performed separate regression models in population 3 for each environment-related variable as the dependent variable and driver’s age and sex as the independent variables. The female-to-male OR for the first comparison was 1.12, but values below 1 were found for extreme age groups. In the second comparison, an OR of 0.50 (0.49–0.51) was found, with progressively lower OR values as age increased. In population 3, women were found to drive less than men in environments known to be high risk (i.e., open roads, night-time, poor light conditions, and weekends). A significant gender gap exists in the amount and type of driving exposure. Although women obtain a driving license more frequently than men, they drive much less and tend to avoid high-risk environments. These results emphasize the need to incorporate a gender perspective in the development and implementation of road safety interventions.

## 1. Introduction

Many studies have addressed sex or gender differences in population-based rates of road crash injury or death, with higher values usually observed in men [1,2,3,4,5,6], specifically in drivers’ risk of road crash or crash-related injury or death. In fact, there is consistent evidence showing increased male-to-female ratios in this context [7,8,9,10,11,12], although this pattern seems to change with age, with higher risk values found in men, especially at younger ages [11,13,14,15,16]. Furthermore, conflicting findings arise when specific issues, such as injury severity, are considered (i.e., depending on whether death or serious injury is considered as the outcome, the risk may be higher for men or for women in different studies) [3,8,17]. One of the main factors that needs to be considered to explain these sex-related differences is driving exposure: the proportion of drivers (i.e., licensed drivers) among the population and the amount of driving exposure (measured in number of trips, or in time or distance driven) among drivers. Most previous studies, conducted in countries as diverse as the UK, the USA, Spain, Denmark, or Sweden describe an increased amount of exposure in male drivers compared to females [3,12,18,19,20,21,22]. Accordingly, in order to compare road crash rates between sexes, most studies adjust their estimates for the amount of driving exposure [3,7,8,9,10,11,23]. However, one common drawback of these studies is the absence or incomplete stratification of this exposure by relevant environmental factors (both spatial and temporal), which are strongly related to the risk of being involved in a road crash, such as the time of day, type of road, or weather conditions, among others. This leads to incomplete adjustment for exposure and, in turn, to an inadequate understanding of the underlying reasons that explain sex differences in drivers’ risk of causing or being involved in a road crash.

To our knowledge, only a few previous studies in Spain aimed to identify sex differences in the amount of driving exposure [24], and none elsewhere focused on analyzing these differences across categories of environmental variables. The limited evidence available on this topic led us to perform the current study, in which the objective was to assess sex-related differences in the amount of driving exposure of car drivers in Spain, from 2014 to 2017, overall and stratified by the main environment-related driving conditions, using a quasi-induced exposure approach.

## 2. Materials and Methods

A retrospective cross-sectional study was performed based on the comparison of sex distributions (overall and stratified by age groups) across three populations in Spain, from 2014 to 2017. These populations included (i) the total number of person-years older than 18 years old (minimum age to obtain a driving license in Spain), obtained from the estimates of the Spanish National Statistics Institute [25]; (ii) the total number of person-years older than 18 years old holding a valid car-driving license, obtained from the Spanish National Register of licensed drivers database of the Spanish Traffic Directorate [26]; and (iii) the total number of car drivers deemed non-responsible for clean collisions in which they were involved, obtained from the Spanish Register of Road Accidents with Victims [27]. We labeled ‘clean collisions’ as those crashes between two or more moving vehicles in which only one of the involved drivers committed a driving error or infraction immediately prior to the crash.

We applied the quasi-induced exposure approach [28]. This method considers that, when analyzing a clean collision, the non-responsible car drivers involved are representative of the entire population of car drivers on the road at the place and time at which the crash occurred (they would be considered as having the “misfortune” to be in the wrong place at the wrong time). The validity of this approach depends on the correct assignation of the car drivers’ responsibility by the police officer in the crash place, as detailed in the Discussion. For this population, information contained in the register was collected on the following environment variables: location of the crash (open roads or urban areas), type of road (highway/motorway, conventional two-lane road, street, other), time of day (12:00 to 5:59 a.m., 6:00 to 11:59 a.m., 12:00 to 5:59 p.m., 6:00 to 11:59 p.m.), day of the week (weekday, weekend), surface condition (normal, altered), weather condition (good, adverse), light conditions (daylight, sunrise/sunset with artificial lighting, sunrise/sunset without artificial lighting, darkness with artificial lighting, darkness without artificial lighting), and traffic density (low, medium, high, very high).

Three consecutive approaches were applied. First, we calculated the female-to-male odds ratios (ORs) between licensed drivers compared to the entire population (overall and stratified by age). Second, we estimated the female-to-male ORs (and the corresponding 95% confidence intervals) between non-responsible car drivers (NRDs) involved in a clean collision (overall and stratified by each environmental or time-related variables) compared to licensed drivers (overall and stratified by age). Finally, in the subpopulation of NRDs, separate multivariate models for each environmental variable (taken as the dependent variable) were performed. In all models, the sex and age of the driver were included as independent variables. The addition of an interaction term between them was tested in all models using the likelihood ratio test (*p* < 0.05). Binomial and multinomial logistic regression models were applied for dichotomous and polytomous dependent variables, respectively. This allowed us to obtain adjusted estimates of the strength of the association (ORs for binary models and relative risk ratios for multinomial ones) between female sex and the probability of driving on each specific environment. Analyses were performed using R (version 3.6.1) [29,30] and Stata (version 15) [31].

## 3. Results

Table 1 shows the distribution of the three study populations according to sex and age strata, as well as the two crude ORs described in the methods.

The female-to-male OR in the population of licensed drivers compared to the overall Spanish population was 1.12. However, striking differences were found across age strata. The female-to-male OR was 0.89 in the youngest age group (18–20 years old). In the subsequent age groups, increasingly higher-than-one OR values were observed, up to a maximum of 1.36 in the 40–44 year old group. In the remaining age groups, the values progressively decreased, reaching a lower-than-one OR value (0.82) in the 65–69 year old group, and the lowest value (0.27) in the oldest age group. In addition, the female-to-male OR in the population of NRDs compared to licensed drivers was 0.50. In the younger age groups (i.e., up to 34 years old), values slightly higher than 0.60 were found. From this age onward, decreasing OR values were observed, with the lowest one (0.21) observed in the group over 74 years old.

Table 2 shows the crude female-to-male ORs in the population of NRDs compared to licensed drivers for each driving environment. All values were lower than 1, ranging from 0.56 (driving in very high traffic density) to 0.35 (driving from 12:00 to 5:00 a.m.). Other low OR values were found for driving without natural or artificial lighting (0.37) and on weekends (0.39). For all driving environments, increasing age was related to lower OR values (data not shown).

Table 3 shows the measures of association (obtained using binary or multinomial logistic regression models) between female sex and selected environment-related driving conditions among the subpopulation of NRDs. For all of them, p-values of the interaction term between sex and age categories were <0.001. Accordingly, the table shows separate estimates for each age group. For drivers under 25 years old, no significant differences between men and women were found for any driving environment. On the other hand, the low number of drivers (especially women) in the older age groups (especially from 70 years old onward) resulted in inaccurate estimates with wide confidence intervals. Regarding the driving area, a significantly lower exposure was observed for female drivers compared to male drivers on open roads between the 35 and 74 year old groups, with a slightly decreasing trend with increasing age. Female drivers tend to drive less than their male counterparts between 6:00 and 11:59 p.m., and even less between 12:00 and 5:59 a.m. Consequently, lower exposure in female drivers was related to driving without natural or artificial lighting. Female drivers in the 25–64 year old age groups also drove significantly less on weekends than their male counterparts. No significant between-sex differences were observed for weather conditions, road surface, and traffic density (data not shown).

## 4. Discussion

A lower driving exposure was estimated among women compared to men in Spain between 2014 and 2017. However, the proportion of licensed drivers was higher among women. Therefore, lower exposure in women can be explained by the fact that, in the subpopulation of people allowed to drive a car (i.e., licensed drivers), females drive much less (approximately half) than men. As we outlined in the introduction, this lower driving exposure in women has been observed in some countries, such as Denmark, the US, and Sweden [12,18,19,21,22]. Interestingly, the same pattern found in our study, i.e., a higher proportion of licensed drivers but a lower distance driven in female drivers compared to males, has been observed in the United States [22].

The higher proportion of female licensed car drivers is reversed in the extreme age groups. Some previous studies in other countries also found that young men are more likely to have a driving license than women [32,33]. This could be explained by a tendency for women to delay the age at which they obtain a driving license compared to men. This delay might also partially explain the higher proportion of licensed drivers among women in the successive age groups, with the largest difference in the 40–44 year old group. These differences decrease after this age, reversing in the 65–69 age group. A cohort effect in the proportion of women who obtain a driving license can explain this trend. Until the late 1960s, driving was almost exclusively for men in Spain, which explains the extremely low proportion of women with a driving license in people over 74 years old between 2014 and 2017. In the following decades, successive cohorts of females progressively started to take up driving. This trend lead to a convergence in the proportion of licensed drivers between men and women over time, a phenomenon described in several countries [18,20,22]. Furthermore, it has been observed that older female drivers renew their driving license less frequently than male drivers of the same age [34], which can also partly explain the low proportion of licensed drivers among older women.

Most authors explain women’s underexposure to car driving based on the overlap of a wide range of economic and social factors: lower affordability to buy a car, less work-related mobility (e.g., shorter commuting distances and times), higher use of alternative modes of transport (as passengers of private and public transport), and higher involvement in activities performed at or near home [12,24,35,36,37,38]. The confluence of male and female roles regarding these issues would explain the aforementioned convergence in mobility patterns over time. On the other hand, a higher level of driving-related stress or anxiety, and a higher level of self-regulation, have also been proposed to explain women’s underexposure to driving [39,40].

Although women of all ages—even the youngest ones—drive less than men of the same age in the population of licensed drivers, the magnitude of this underexposure increases with age. This result is consistent with previous studies showing decreased and even reversed differences in exposure to driving between men and women in younger cohorts of drivers [20,41]. In line with our results, a slightly higher proportion of men than women drove 10 or more hours per week in the DRIVE cohort of young drivers in Australia [8]. Additionally, it has been observed that among older drivers, women are more likely to voluntarily reduce or stop driving [35,42,43,44].

The results in Table 2 reveal that, in the population of licensed car drivers, the underexposure of female drivers on the road is preserved for all driving environments. However, there are notable differences depending on the specific driving environment. In fact, in the subgroup of NRDs, which is representative of the drivers on the road (Table 3), a higher relative exposure of women was observed in urban areas, during daytime (from 6:00 a.m. to 5:59 p.m.), good light conditions, and weekdays. This pattern is generally observed in middle-age groups (i.e., 25–64 years old): no differences were found for younger drivers and the low sample size does not allow us to detect differences in the older age groups. Again, several hypotheses may explain these findings. For instance, the higher relative exposure in urban areas and during daytime in middle-aged female drivers may be related to certain types of trips that women make more frequently (e.g., short-distance commuting trips, transporting children to school and other activities, transporting adults to medical appointments, shopping) [12,19,21,36,37,45]. Conflicts between work and family responsibilities may explain the low exposure of women to driving on work-related or long-distance travels (i.e., on open roads) [18].

On the other hand, our results show that most environments in which the relative exposure of female drivers is higher are a priori related to a lower risk of crash-related fatalities or severe injuries (e.g., urban areas, daytime, good light conditions, weekdays, or good infrastructure) [46,47,48,49,50,51,52]. Accordingly, several previous studies have found a higher degree of self-regulation (i.e., restricting driving exposure to avoid risky driving circumstances) in women, especially among older ones, perhaps due to less driving experience and self-confidence or higher driving-related anxiety [34,35,39,44].

Apart from its descriptive nature, our study has other limitations that should be considered. Some of them are related to one of the data sources: the Spanish Register of Road Accidents with Victims is a police-based registry in which most severe crashes are over-represented. In addition, the collection of some subjective variables might be dependent on the police officer. Additionally, previous studies [53,54] have reported that in this type of register, crashes involving women are slightly less likely to be reported compared to those involving men. This could be explained by the fact that women tend to drive more frequently in urban settings and usually have more cautious driving styles, and both factors are associated with minor accidents (which are under-represented in police-based registries). Besides, some interesting variables, such as the reasons for driving, are poorly collected or even absent. In particular, for the purpose of our study, information on driver gender (in addition to driver sex) would have been invaluable, but this variable is missing in the register. Other limitations are linked to our application of the quasi-induced exposure approach, in particular with regard to the method followed to assign crash responsibility (and therefore to identify the subpopulation of non-responsible drivers) [55], and the validity of the assumptions by which these NRDs can be considered representative of the entire population of drivers on the road. The latter issue has been extensively discussed in previous works by our research team and other authors [56,57,58,59,60]. Briefly, the validity of this method depends on the correct identification of responsibility by the police officers (based on the commission of infractions). Therefore, an incorrect recording of driver infractions—independent of driver characteristics—would lead to a toward-the-null bias.

## 5. Conclusions

Our results reveal that even nowadays in a developed country, such as Spain, and even in the youngest age groups, an important gender gap exists with regard to the amount and type of exposure to driving. Overall, the observed pattern (lower exposure in women, especially in driving environments where the risk of severe crash-related injuries is higher) undoubtedly contributes to the explanation of the lower crash injury and crash death rates usually observed in women in most previous investigations. Nevertheless, the possible implications of other gender-dependent behavioral differences cannot be discarded. These findings emphasize the need to incorporate a gender perspective in the development and implementation of road safety interventions in our country [21,61]. It is also necessary to conduct further studies aimed at identifying the reasons for these sex-related differences, which may differ depending on the context. In this sense, qualitative approaches may be useful to obtain this information. Although the following quote by Polk was written in the context of climate change, it is also valid in the field of road safety: ‘the travel habits and attitudes which dominate among women should also be seen as a norm within the transport sector, and integrated more fully into planning and policy’ [62].

## Figures and Tables

**Table 1:** Distribution of the three populations according to sex and age. Female-to-male ORs between populations.

	Persons-Years of Spanish Population ≥18 y.o.		Person-Years of Licensed Car Drivers (≥18 y.o.)		NRDs * Involved in Clean Collisions		Licensed Drivers/Population	NRDs/Licensed Drivers	
Age	Males	Females	Males	Females	Males	Females	OR	OR	95% CI
All ages	72,845,320	76,210,495	34,152,837	40,096,817	32,684	19,145	1.12	0.50	(0.49–0.51)
18 to 20	2,689,048	2,540,808	842,219	705,390	928	463	0.89	0.60	(0.53–0.67)
21 to 24	3,754,844	3,618,528	1,864,948	2,054,996	2129	1468	1.14	0.63	(0.59–0.67)
25 to 29	5,135,457	5,100,161	2,968,105	3,575,295	3360	2547	1.21	0.63	(0.60–0.66)
30 to 34	6,090,909	6,094,552	3,575,153	4,462,014	3755	2839	1.25	0.61	(0.58–0.64)
35 to 39	7,634,711	7,459,244	4,372,787	5,493,925	4488	3183	1.29	0.56	(0.54–0.59)
40 to 44	8,009,656	7,738,559	4,157,080	5,473,925	4213	2917	1.36	0.53	(0.50–0.55)
45 to 49	7,528,746	7,386,104	3,769,103	4,940,334	3535	2174	1.34	0.47	(0.44–0.49)
50 to 54	6,974,329	7,012,825	3,284,355	4,238,550	2947	1526	1.28	0.40	(0.38–0.43)
55 to 59	6,111,803	6,297,199	2,814,420	3,579,022	2477	992	1.23	0.31	(0.29–0.34)
60 to 64	5,066,409	5,360,808	2,325,983	2,551,835	1756	520	1.04	0.27	(0.24–0.30)
65 to 69	4,513,768	4,968,627	1,934,249	1,741,161	1372	300	0.82	0.24	(0.21–0.28)
70 to 74	3,751,614	4,355,627	1,186,658	851,112	907	145	0.62	0.22	(0.19–0.27)
>74	5,584,026	8,277,453	1,057,777	429,258	817	71	0.27	0.21	(0.17–0.27)

* NRDs: non-responsible car drivers.

**Table 2:** Female-to-male ORs in the population of 103,658 non-responsible car drivers for each driving environment compared to licensed drivers.

Variable	Categories	OR	95% CI
Area	Urban	0.55	(0.53–0.56)
	Open roads	0.47	(0.45–0.48)
Type of road	Highway/Motorway	0.49	(0.47–0.51)
	Conventional two-lane roads	0.46	(0.44–0.47)
	Streets	0.55	(0.53–0.57)
	Other	0.47	(0.43–0.51)
Time of day	12:00 to 5:59 a.m.	0.35	(0.32–0.39)
	6:00 to 11:00 a.m.	0.54	(0.52–0.56)
	12:00 to 5:59 p.m.	0.53	(0.52–0.55)
	6:00 to 11:59 p.m.	0.44	(0.42–0.45)
Type of day	Weekday	0.53	(0.52–0.54)
	Weekend	0.39	(0.37–0.40)
Road Surface	Normal	0.50	(0.49–0.51)
	Altered	0.49	(0.46–0.51)
Weather conditions	Good	0.50	(0.49–0.51)
	Adverse	0.49	(0.47–0.51)
Light conditions	Daylight	0.52	(0.51–0.53)
	Sunrise/sunset with artificial lighting	0.48	(0.44–0.53)
	Sunrise/sunset without artificial lighting	0.48	(0.43–0.54)
	Darkness with artificial lighting	0.44	(0.42–0.47)
	Darkness without artificial lighting	0.37	(0.35–0.40)
Traffic density	Low	0.47	(0.46–0.48)
	Medium	0.53	(0.51–0.55)
	High	0.54	(0.51–0.56)
	Very high	0.56	(0.50–0.63)

**Table 3:** Association of being a female among 103,658 non-responsible car drivers for selected environment-related driving conditions.

	Driving on Open Roads (Ref.: Urban Areas)		Driving from 12:00 to 5:59 a.m. (Ref.: 6:00 to 11:59 a.m.)		Driving from 6:00 to 23:59 p.m. (Ref.: 6:00 to 11:59 a.m.)		Driving without Natural or Artificial Light (Ref.: Daylight)		Driving on Weekends (Ref: Weekday)	
Age	OR	95% CI	RRR	95% CI	RRR	95% CI	RRR	95% CI	OR	95% CI
18 to 20	1.24	(0.99–1.55)	1.06	(0.64–1.76)	1.15	(0.89–1.49)	0.73	(0.47–1.12)	0.90	(0.69–1.17)
21 to 24	0.98	(0.86–1.12)	0.76	(0.56–1.04)	0.86	(0.73–1.00)	0.77	(0.60–1.00)	0.92	(0.79–1.08)
25 to 29	0.97	(0.88–1.08)	0.70	(0.53–0.91)	0.77	(0.68–0.87)	0.68	(0.56–0.83)	0.71	(0.63–0.81)
30 to 34	0.94	(0.85–1.04)	0.50	(0.37–0.69)	0.75	(0.67–0.85)	0.63	(0.52–0.76)	0.68	(0.61–0.77)
35 to 39	0.88	(0.81–0.97)	0.54	(0.39–0.74)	0.71	(0.63–0.79)	0.60	(0.50–0.72)	0.71	(0.63–0.79)
40 to 44	0.80	(0.73–0.88)	0.56	(0.41–0.78)	0.78	(0.69–0.87)	0.61	(0.50–0.73)	0.72	(0.64–0.81)
45 to 49	0.79	(0.71–0.88)	0.70	(0.49–0.98)	0.87	(0.76–0.99)	0.76	(0.61–0.94)	0.71	(0.62–0.82)
50 to 54	0.86	(0.76–0.98)	0.42	(0.26–0.71)	0.81	(0.69–0.94)	0.79	(0.61–1.02)	0.70	(0.60–0.81)
55 to 59	0.83	(0.71–0.96)	0.30	(0.16–0.55)	0.76	(0.64–0.92)	0.66	(0.49–0.89)	0.66	(0.55–0.80)
60 to 64	0.74	(0.61–0.90)	0.64	(0.32–1.29)	0.73	(0.57–0.94)	0.88	(0.58–1.33)	0.76	(0.59–0.96)
65 to 69	0.66	(0.52–0.86)	1.37	(0.57–3.27)	0.95	(0.70–1.29)	0.99	(0.61–1.60)	0.91	(0.67–1.22)
70 to 74	0.54	(0.38–0.78)	1.24	(0.14–10.82)	0.86	(0.55–1.34)	0.44	(0.16–1.23)	0.70	(0.45–1.10)
>74	0.80	(0.48–1.34)	1.37	(0.17–11.11)	1.19	(0.61–2.34)	1.12	(0.39–3.24)	1.18	(0.68–2.05)

## Data Availability

The data underlying this article were provided by the Spanish Traffic Directorate with permission. Data will be shared upon request to the corresponding author with permission of the Spanish Traffic Directorate.
